# Worldwide disparities in access to treatment and investigations for nephropathic cystinosis: a 2023 perspective

**DOI:** 10.1007/s00467-023-06179-3

**Published:** 2023-11-18

**Authors:** Maitena Regnier, Sacha Flammier, Mounia Boutaba, Aliou Abdoulaye Ndongo, Aude Servais, Franz Schaefer, Elena Levtchenko, Justine Bacchetta, Aurélia Bertholet-Thomas

**Affiliations:** 1 Centre de Référence Des Maladies Rénales Rares Néphrogones, Hôpital Femme–Mère–Enfant, Hospices Civils de Lyon & Université Claude-Bernard, Lyon 1, Lyon, France; 2 Service de Néphrologie, Rhumatologie Et Dermatologie Pédiatriques, Hôpital Femme Mère Enfant, Hospices Civils de Lyon, Boulevard Pinel, 69677 Bron Cedex, France; 3 Faculté de Médecine Lyon Est, Université Claude Bernard, Lyon 1, Lyon, France; 4 Department of Pediatrics A, Hussein Dey University Hospital Center, University of Algiers 1, Algiers, Algeria; 5 Pediatric Unit, Aristide Le Dantec Hospital Cheikh Anta Diop University of Dakar, Dakar, Senegal; 6 Service de Néphrologie Et Maladies Métaboliques Adulte Hôpital Necker 149, Paris, France; 7 Division of Pediatric Nephrology, University Children’s Hospital Heidelberg, Heidelberg, Germany; 8 International Pediatric Nephrology Association (IPNA), C/o Nationwide Children’s Center for Faculty Development (ED-5081), 700 Children’s Drive, Columbus, OH 43205 USA; 9 European Rare Kidney Disease Reference Network (ERK-Net) Project Office, Im Neuenheimer Feld 130.3, D-69120 Heidelberg, Germany; 10 Division of Pediatric Nephrology, Department of Pediatrics, University Hospitals Leuven, University of Leuven, Leuven, Belgium; 11 ORKID : Filière Orphan Kidney Diseases, Montpellier, France; 12 Diagnostic Et Traitements Des Maladies Osseuses, INSERM 1033 Physiopathologie, Paris, France

**Keywords:** Nephropathic cystinosis, Cysteamine availability, Developing economies, Worldwide disparities

## Abstract

**Background:**

Nephropathic cystinosis (NC) is a rare lysosomal disease, leading to early kidney failure and extra-renal comorbidities. Its prognosis strongly relies on early diagnosis and treatment by cysteamine. Developing economies (DEing) face many challenges when treating patients for rare and chronic diseases. The aim here is to evaluate the access to investigations and treatment in DEing, and to assess for potential inequalities with Developed Economies (DEed).

**Methods:**

In this international cross-sectional study, a questionnaire on access, price and reimbursement of genetic, biological analyses, and treatment was sent to nephrology centers worldwide during 2022.

**Results:**

A total of 109 centers responded, coming from 49 countries and managing 741 patients: 43 centers from 30 DEing and Economies in transition (TrE), and 66 from 19 DEed. In 2022, genetics availability was 63% in DEing and 100% in DEed, whereas intra leukocytes cystine levels (IL-CL) were available for 30% of DEing patients, and 94% of DEed patients, both increasing over the last decade, as has access to immediate release cysteamine and to cysteamine eye drops in DEing. However, delayed released cysteamine can be delivered to only 7% vs. 74% of patients from DEing and DEed, respectively, and is still poorly reimbursed in DEing.

**Conclusions:**

Over the last decade, access to investigations (namely genetics and IL-CL) and to cysteamine have improved in DEing and TrE. However, discrepancies remain with DEed: access to delayed released cysteamine is limited, and reimbursement is still profoundly insufficient, therefore limiting their current use.

**Graphical abstract:**

**Figure:**
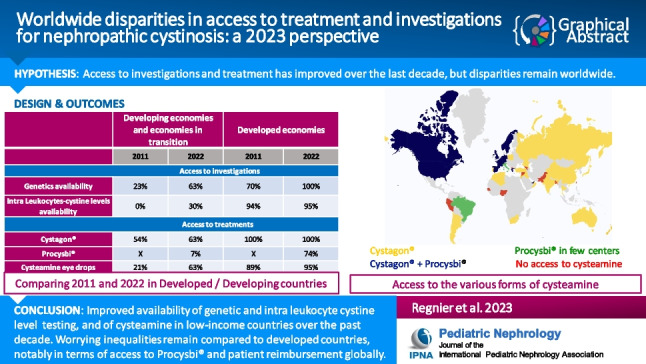
A higher resolution version of the Graphical abstract is available as Supplementary information

**Supplementary Information:**

The online version contains supplementary material available at 10.1007/s00467-023-06179-3.

## Introduction

Nephropathic cystinosis (NC) is a rare autosomal recessive lysosomal storage disease [1], due to pathogenic variants in the *CTNS* gene [2]. Its estimated incidence ranges from 0.5 to 1 per 100,000 live births in developed nations [3]. The *CTNS* gene encodes cystinosin, the lysosomal cystine transporter, the impairment of which leads to systemic intra-lysosomal cystine accumulation, resulting in systemic tissue damages [4]. In the most common and severe form of the disease, patients suffer from complete proximal tubulopathy, named renal Fanconi syndrome, and progress to kidney failure during the first decade of life [5]. Other organs are also affected including the eyes, thyroid, bones, muscles, pancreas, and gonads [6–9]. The diagnosis can be made by detecting either elevated intra-leukocytes cystine levels (IL-CL), or cystine crystals in the cornea on slit lamp examination, confirmed by genetic testing if possible [10]. The introduction of the cystine-depleting agent cysteamine in the 1980s remarkably improved the prognosis of NC, delaying both the progression to kidney failure and the onset of other extra-renal complications [11, 12], and improving linear growth if initiated at an early age [13]. Since then, several studies demonstrated that higher mean IL-CL and delayed initiation of treatment are significant risk factors for early progression to kidney failure and poor linear growth [14]. Hence, access to monitoring and early treatment is critical for the prognosis of NC patients.

Developing Economies (DEing) face many global challenges when treating children with complex and rare kidney diseases [15], including NC, as demonstrated in our previous survey conducted in 2011 [16, 17]. In this study, we pooled data from 213 patients, followed in 41 centers from 30 countries, including 109 patients from Developed Nations and 104 from Developing Nations. At that time, we showed major discrepancies between Developed and Developing Nations, both in terms of access to investigations and to treatment. This resulted in poorer outcomes in Developing Nations, including shorter life expectancy, earlier kidney failure with 50% lower median kidney survival rate, and inferior linear growth [17]. These findings are consistent with a recently published very large cohort, describing age at initiation of cysteamine as a significant risk factor for poor linear growth [14].

Over the last decade, new therapies such as delayed-released cysteamine have emerged [18]. Thus, we aimed to update the current status of patients with NC worldwide, focusing on diagnosis and access to treatment, in order to highlight persistent territorial disparities and to try to provide health care providers with practical tools when negotiating with private insurance and public health systems.

## Methods

Our survey, an international cross-sectional study utilizing a Google form, included 43 general items on demographics, management strategy, access, pricing, and reimbursement of investigations (i.e., genetics and IL-CL) and treatment (i.e., cysteamine, formulation, and daily dose adjustment; cysteamine eye drops; access to dialysis and transplantation; access to recombinant human growth hormone rhGH), and access to transition programs and multi-disciplinary care. The reimbursement of investigations and treatment was asked regarding whether it was total, partial, or zero. The questionnaire did not include any patient data and thus did not require any ethical approval. It was sent between January 2022 and September 2022 by email to nephrology centers worldwide (pediatric and adults), using different mailing lists from the International Pediatric Nephrology Association (IPNA), the European Society for Pediatric Nephrology (ESPN), the European Rare Kidney Disease Reference Network (ERK-Net), the Cystinosis Research Foundation, the international PedNeph email server (pedneph-request@lists.uchicago.edu), the African mailing list of pediatric nephrologists, and miscellaneous centers and nephrology authors worldwide. The original survey is presented in Supplemental File 1. In case of inconsistent answers from centers coming from the same country, we directly contacted the centers to clarify the differences.

It should be noted that this survey was also supported (and strongly suggested) by some patient associations, notably Cystinosis Ireland: they wanted to get data from physicians from different countries to get a clearer picture of the reimbursement status of the different forms of cysteamine in different countries.

To compare high- and low-income countries, DEed, DEing, and Economies in Transition (TrE) were defined according to the last 2022 United Nations country classification [19]. Since we received only four answers from TrE, which cannot be representative of this category, we decided to combine DEing and TrE in a common group. World maps were designed online on the free website Visme®.

For statistical analysis, the categorical variables were expressed as number (*N*) and percentage. Categorical variables were compared using the chi-square test or Fisher’s exact test if the conditions of application of Chi square test were not met. Quantitative variables were expressed as median (minimum–maximum). Quantitative variables were compared between groups using Student’s *t* test after verification of equality of variances when data were normally distributed, and with the nonparametric Wilcoxon test statistics when the hypothesis of normality of distribution was not verified. The statistical tests were bilateral and the level of significance was set to 5% (*p* < 0.05). Statistical analyses were conducted using the online website BiostatsTGV®.

## Results

In total, 66 centers coming from 19 DEed, 4 centers coming from 4 TrE, and 39 centers coming from 26 DEing answered, reporting a total of 741 patients to be followed in these centers (462 from DEed, 6 from TrE, and 273 from DEing), as summarized in Table 1. Of note, 94% of the respondents were pediatric nephrologists.

**Table 1:** Number of answering centers with their respective number of pediatric and adult patients, classified using the 2022 Classification of the United Nations

Developed economies					Developing economies					
Country (number of centers)			Total of patients, *N* (pediatrics + adults)		Country (Number of centers				Total of patients, *N* (pediatrics + adults)	
Australia	(3)		9	(9 + 0)	Algeria		(1)		6	(6 + 0)
Belgium	(3)		9	(4 + 5)	Argentina		(1)		2	(2 + 0)
Canada	(3)		20	(20 + 0)	Bahrain		(1)		7	(5 + 2)
France	(11)		94	(35 + 59)	Bangladesh		(1)		1	(1 + 0)
Germany	(5)		143	(67 + 76)	Brazil		(4)		26	(26 + 0)
Greece	(1)		2	(2 + 0)	Chile		(1)		6	(4 + 2)
Italy	(4)		52	(40 + 12)	China		(1)		8	(8 + 0)
Japan	(1)		3	(1 + 2)	Ecuador		(2)		3	(3 + 0)
Luxembourg	(1)		0	0	India		(3)		6	(6 + 0)
Netherlands	(4)		23	(14 + 9)	Iraq		(1)		5	(5 + 0)
New Zealand	(1)		9	(5 + 4)	Israel		(1)		7	(2 + 5)
Norway	(1)		5	(3 + 2)	Jordan		(1)		5	(3 + 2)
Poland	(3)		3	(3 + 0)	Lebanon		(1)		4	(4 + 0)
Portugal	(1)		3	(3 + 0)	Morocco		(2)		7	(6 + 1)
Spain	(7)		26	(25 + 1)	Nigeria		(1)		0	0
Sweden	(1)		4	(4 + 0)	Oman		(1)		2	(2 + 0)
Switzerland	(2)		3	(3 + 0)	Pakistan		(1)		4	(4 + 0)
United Kingdom	(1)		14	(14 + 0)	Peru		(1)		0	0
USA	(13)		40	(33 + 7)	Senegal		(1)		4	(2 + 2)
					South Africa		(4)		33	(30 + 3)
					Syrian Republic		(1)		9	(9 + 0)
					Taiwan		(1)		2	(0 + 2)
					Thailand		(1)		0	0
					Tunisia		(1)		9	(8 + 1)
					Turkey		(4)		113	(94 + 19)
					United Arab Emirates		(1)		4	(4 + 0)
TOTAL										
19 countries 66 centers			462 (285 + 177)		26 countries 39 centers				273 (234 + 39)	
**Economies in transition**										
Countries (number of centers, *N*)						Total of patients *N* (pediatrics + adults)				
Armenia		(1)				0		0		
Georgia		(1)				1		(1 + 0)		
North Macedonia		(1)				0		0		
Russian Federation		(1)				5		(5 + 0)		
TOTAL										
4 countries, 4 centers						6 (6 + 0)				

**Fig. 1:**
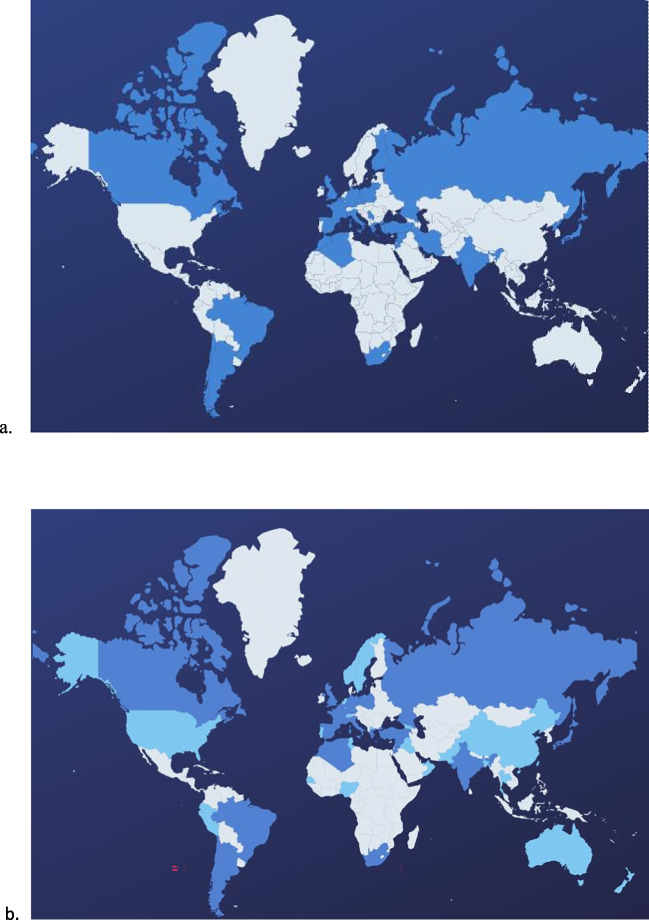
Responding countries in 2011 and in 2022. **a** Answers from 2011: 40 centers from 30 countries. A total of 213 patients followed. **b** Answers from 2022: 109 Centers from 49 Countries. A total of 741 patients followed. In dark blue, countries which had answered in 2011. In light blue, new answering countries

Compared to the 2011 survey, we obtained answers from more countries, as illustrated in Fig. 1. The number of patients followed in 2011 and in 2022 also increased, thus allowing us to have a better view on cystinosis management around the world. Almost all the answering countries of 2011 also responded to the current survey. We compared the answering centers of DEing/TrE for both surveys, and they were identical for Moscow (Russia), Skopje (North Macedonia), Yerevan (Armenia), Buenos Aires (Brazil), Algiers (Algeria), Ankara (Turkey), Damascus (Syria), Casablanca (Morocco), and Beirut (Lebanon). For the last three centers, the answers came from the same physician in 2011 and 2022. The most relevant items are synthetized in Table 2, for the TrE and DEing on the one hand, and for the DEed on the other hand; the answers to the other items are all displayed in Supplemental Table 1, notably the availability and reimbursement of other formulations of cysteamine, the price and reimbursement policy of rhGH, and the cysteamine eye drops formulation available around the world.

**Table 2:** Answers to the survey for DEing/TrE and for DEed

**a. Demographic features of patients followed in the different centers**						
		Developing economies and economies in transition		Developed economies		*p* value
		*N* = number	*N* (%)	*N* = number	*N* (%)	
Demographic features						
Number of patients		279	100	462	100	
Number of pediatric patients		240	86	285	61.7	*p* < 0.0001
	Conservative Treatment	178	74.2	232	81.4	*p* = 0.042
	Dialysis	23	9.6	12	4.2	*p* = 0.021
	Kidney transplant	38	25.8	39	13.7	*p* = 0.53
Number of adult patients		39	14	177	38.3	*p* < 0.0001
	Conservative Treatment	10	25.6	35	19.8	*p* = 0.39
	Dialysis	6	15.4	21	11.9	*p* = 0.59
	Kidney transplant	23	59	122	68.9	*p* = 0.26
**b. Access to investigations and treatment by countries**						
		Developing economies and economies in transition		Developed economies		*p* value
		*N*	*N*(%)/median [min;max]	*N*	*N*(%)/median [min;max]	
Access to investigations, *N* = Number of countries						
Genetics availability		19	63.3	19	100	0.0034
Price/patient (USD)		13	650 [179–1300]	7	780 [275–1200]	*p* = 0.87
Reimbursement	Total	7	36.8	15	78.9	0.02
	Partial	5	26.3	3	15.8	0.69
	None	7	36.8	1	5.3	0.04
Intra leukocyte cystine levels availability		9	30	18	94.7	< 0.0001
Price/patient		5	150 [52–2000]	6	126.5 [78–316]	*p* = 0.66
Reimbursement	Total	4	44.4	16	88.9	0.02
	Partial	3	33.3	1	5.6	0.09
	None	2	22.2	1	5.6	0.25
Indication	To follow patients on cysteamine therapy	4	19	2	10.5	0.66
	To confirm the diagnosis	2	10.5	2	10.5	1
	Both	15	71.4	15	78.9	0.72
Access to treatment, *N* = number of countries						
Cysteamine availability		19	63.3	19	100	0.0033
Cysteamine formulation	Cystagon ®	15	50	19	100	< 0.0001
	Cystagon® + Procysbi ®	2	6.7	14	73.7	< 0.0001
	Other formulations	2	6.7	0	0	0.51
Price of Cystagon® (USD)/gram		4	27.15 [16.5–44]	11	17.95 [1.75–19]	*p* = 0.14
Cystagon® reimbursement	Total	8	42.1	17	89.5	*p* = 0.005
	Partial	5	26.3	2	10.5	*p* = 0.40
	None	6	31.6	0	0	*p* = 0.020
Price of Procysbi ® (USD)/gram		1	1530	6	383.15 [264–698]	NA
Procysbi ® reimbursement	Total	0	0	9	64.3	*p* = 0.175
	Partial	1	50	4	28.6	*p* = 1
	None	1	50	1	7.1	*p* = 0.23
Daily dose adjustment of cysteamine	IL-cystine levels	1	5.6	7	38.9	*p* = 0.041
	BMI	11	61.1	1	5.6	0.0009
	Both	6	33.3	10	55.6	*p* = 0.31
Eye drops availability		19	63.3	18	94.7	*p* = 0.017
Eye drops formulation	Cystadrops®	16	84.2	15	83.3	1
	Cystaran®	1	5.2	0	0	1
	Others formulations available	2	10.5	3	16.7	0.66
Eye drops price	Price per vial (USD)	5	650 [78–1150]	7	1177 [990–1573]	*p* = 0.018
Eye drops reimbursement	Total	7	38.9	16	88.9	*p* = 0.0045
	Partial	4	22.2	1	5.6	*p* = 0.34
	None	7	63.6	1	5.6	*p* = 0.04
**c. Number of centers with easy access to other supplementary care**						
		Developing economies and economies in transition		Developed economies		*p* value
		*N*	*N* (%)	*N*	*N* (%)	
Access to various programs, *N* = Number of centers						
Centers with easy access to:	Hemodialysis	34	82.9	66	100	< 0.001
	Peritoneal dialysis	33	80.5	66	100	*p* = 0.0003
	Kidney transplant	24	58.5	66	100	< 0.0001
	Transition from childhood to adulthood care	11	26.8	44	69.8	< 0.0001
	Multi-disciplinary approach or clinic	8	19.5	33	50	*p* = 0.002

**Fig. 2:**
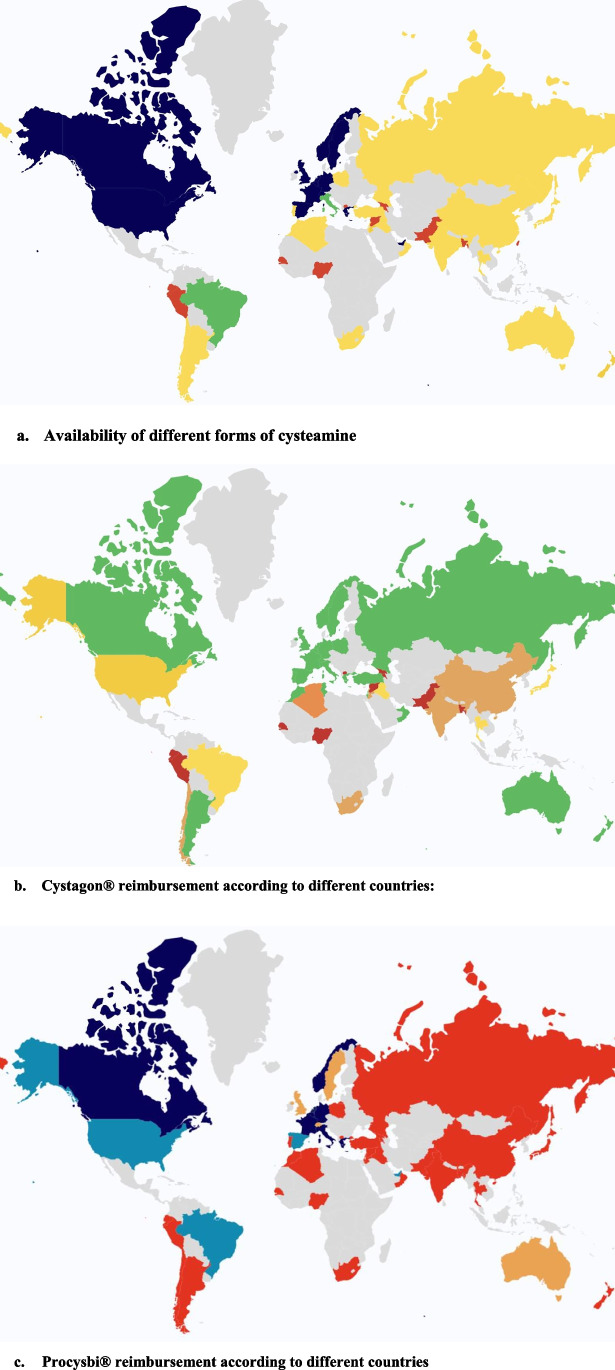
Access to cysteamine (and reimbursement) in 2022. **a** Availability of different forms of cysteamine. In dark blue, countries where both immediate-released form and delayed form of cysteamine are available. In green, countries where immediate-released form is available, with some centers offering delayed-form of cysteamine. In yellow, countries where only immediate-released form of cysteamine is available. In red, countries where no forms of cysteamine can be delivered. In gray, non-responding countries. **b** Cystagon® reimbursement according to different countries: In green, countries with full reimbursement of Cystagon**®**. In yellow, countries with partial reimbursement of Cystagon**®**. In orange, countries which do not reimburse for Cystagon**®**. In red, countries where Cystagon**®** is not available. In gray, non-responding countries. **c** Procysbi® reimbursement according to different countries. In dark blue, countries with full reimbursement for Procysbi**®**. In light blue, countries with partial reimbursement for Procysbi**®**. In orange, countries which do not reimburse for Procysbi**®**. In red, countries where Procysbi**®** is not available. In gray, non-responding countries

In total, 279 patients came from DEing/TrE and 462 patients from DEed, with more adult patients in DEed: 14% vs. 38% (*p* < 0.0001). The adult patients have comparable kidney evolution as they are similarly distributed between “conservative treatment,” “dialysis,” and “kidney transplantation.” As for children, fewer DEing/TrE patients had a functioning native kidney at the time of the study, 74% vs. 81% (*p* = 0.042), with an increased proportion of patients undergoing maintenance dialysis: 10% vs. 4% (*p* = 0.013). Access to genetic screening is 63% in DEing/TrE and 100% in DEed (*p* = 0.0035), whereas intra-leukocytes cystine level testing is available for 30% of DEing patients, as compared to 95% of DEed patients (*p* < 0.0001). Regarding cysteamine treatment, oral cysteamine is available for 63% of patients in DEing/TrE, and for 100% of DEed (*p* = 0.0016); and cysteamine eye drops can be prescribed in 63% in DEing/TrE and 95% in DEed (*p* = 0.016). However, delayed-release of cysteamine can be delivered to only 7% vs. 74% of patients from DEing/TrE and DEed, respectively (*p* = 0.0002). Of note, only two countries prescribe cysteamine formulations other than Cystagon***®*** and Procysbi***®***: Argentina uses cystam “bitrartro de cysteamine,” and some centers in South Africa use cysteamine powder. Figure 2 illustrates the availability of the different formulations of cysteamine in 2022 in different countries, as well as the reimbursement policies for these different compounds.

In total, 83% of DEing/TrE centers declare having easy access to hemodialysis, 81% to peritoneal dialysis, and 59% of them to kidney transplantation, compared to 100% in DEed for each category. Lastly, 27% of DEing/TrE centers organize programs of transition from childhood to adulthood compared to 70% in DEed (*p* < 0.0001), and 20% have formalized multi-disciplinary approaches, vs. 50% in DEed (*p* = 0.002).

Finally, Table 3 compares data obtained in the 2011 and the 2022 surveys, mainly for access to laboratory assessments (genetics and IL-CL) and treatment. The access to genetics has improved in all countries: 23% in 2011 vs. 63% in 2022 (*p* < 0.0001) for DEing/Tr, and 70% vs. 100%, respectively, for DEed (*p* = 0.024). Access to IL-CL has also improved in DEing/TrE: 0% vs. 55% (*p* < 0.0001). As for the evolution of access to treatment, the access to oral cysteamine has not significantly changed, but treatment with cysteamine eye drops has expanded: 21 vs. 63% (*p* < 0.0001). In DEed, the adult living proportion of patients has increased from 26 to 38% (*p* = 0.018) over the last decade.

**Table 3:** Comparison between 2011 and 2022

Variables	Developing economies and economies in transition		*p*	Developed economies		*p*
	2011	2022		2011	2022	
Proportion of adult living patients	14%	14%	1	26%	38%	*p* = 0.018
Access to investigations						
Genetics availability	23%	63%	*p* < 0.0001*	70%	100%	*p* = 0.024
Intra leukocytes-cystine levels availability	0%	30%	*p* < 0.0001*	94%	95%	1
Access to treatments						
Cysteamine	53.8%	63%	*p* = 0.52	100%	100%	1
Eye drops	21%	63%	*p* < 0.0001*	89%	95%	*p* = 0.69

## Discussion

Due to a major effort of many nephrologists who completed the survey, we are able to propose a global worldwide view on the management of patients with NC in 2022, even though we unfortunately still miss data from many countries, in particular coming from Africa, parts of Oceania, Central and South East Asia, and Central America and parts of South America. The current study reveals persistent discrepancies in the management of patients with NC around the world: although the access to laboratory investigations and treatment in DEing and TrE have improved over the last decade, some inequalities remain, especially for the reimbursement of diagnostic tools and treatments, thus obviously contributing to limited access to care for these patients.

As demonstrated in a recent large European paper coming from 9 European countries and Turkey [14], NC outcomes have truly evolved over the last decades. Previously fatal during childhood, NC has become a treatable disease with patients surviving to adulthood with an improved kidney survival rate, notably since the introduction of cysteamine. Nevertheless, this series provides data mainly about disease evolution in DEed countries but not in DEing.

Back to 2011, the year of our first survey [17], there were very little data on management of patients with NC in DEing. Since then, a few reports were published. Some papers enlighten persistent difficulties. For example, in 2018, a case report of two brothers with NC living in China and having no access to cysteamine treatment was published, seeking help from international organizations to obtain the drugs [20]. Since 2018, China seems to have access to cysteamine, as appears from the current survey. Also, in 2018, a group from Chile reported the country’s first case of genetic confirmation of NC [21], demonstrating the progress in diagnostic management in some countries.

Notably, in our survey, we observed a growing number of adult patients in DEed. Indeed, 38% of the current DEed patients are adults, compared with 27% in 2011. The growing number of adult patients may reflect a better “management” of NC and reflect the long-term effects of cysteamine treatment that was initiated since the mid-1980s [11]. However, this may also be due to a higher proportion of adult centers responding to the 2022 survey. Nevertheless, this trend has also been described in other cohorts: for example ECYSCO, a European multicenter longitudinal cohort of the RaDiCo program, including 239 patients with NC, described the proportion of adult patients as 53.9% [22]. The Rare Disease Cohorts Programme “RaDiCo” is coordinated by the French Institut of Health and Medical Research (INSERM). In contrast, the proportion of adult patients in TrE and DEing remained stable (14% in 2011 and 2022). It can obviously be a bias due to the fact that in TrE and DEing, we mainly contacted pediatric nephrologists, but it could unfortunately also be due to a shorter life expectancy of patients in TrE and DEing, reflecting years of insufficient access to treatment and monitoring. Indeed, the proportion of pediatric patients still having functioning native kidneys is smaller in TrE/DEing than in DEed, probably due to insufficient access to cysteamine, known to be a major risk factor of progression to kidney failure [14]. Moreover, the proportion of adult patients (14%) is consistent with figures previously observed in European historical untreated cohorts [23]; we can assume this number will increase in the future, as it did in DEed with the improvement in care for these patients. On the other hand, the current proportion of patients with kidney transplant is similar worldwide, both in pediatric and in adult patients. It hopefully reflects a global improvement in access to kidney transplantation, as shown by the proportion of TrE/DEing countries declaring having easy access to transplantation (61%). Patients living in those countries probably also benefit from better access to dialysis: 83% of centers declare having easy access to hemodialysis (HD) or peritoneal dialysis (PD).

Overall, we describe a global improvement in access to treatment and investigations for NC in DEing over the last decade: genetics is available in 63% of DEing and 100% of DEed, whereas IL-CL is available for 55% of DEing/TrE patients, and for 94% of DEed patients. This represents a major improvement in DEing/TrE compared with 2011, when genetics was performed in 23% of the patients, and IL-CL were only mentioned for 2 patients. IL-CL is a rather challenging biological analysis: methods vary from one laboratory to another, requiring standardized norms for each laboratory; instruments and techniques are sophisticated, restricting their availability to few centers, even in DEed; and it is even further complicated by the sample sensitivity to storage and transport conditions. All these analytical and pre-analytical challenging steps complicate its extension to numerous centers, and make the increase in accessibility of this assessment since 2011 very impressive.

In DEing and TrE, the daily dose adjustment is still largely based on anthropometry. Hopefully, in the future, easier and cheaper access to IL-CL can be applied for routine adjustment of cysteamine dose, as recommended by the latest KDIGO conference [24].

Access to systemic cysteamine for patients from DEing has also improved over the last decade: from 53 to 63%. Nonetheless, the gap to the situation in DEed, where all patients are treated with cysteamine, is still substantial and even more pronounced for delayed release cysteamine, which can only be delivered to 7% vs. 71% of patients from DEing/TeE and DEed, respectively. The DEing also appear to catch up regarding the use of eye drops, which has increased from 21 to 63% as compared to a change from 89 to 95% in DEed.

Lastly, even though diagnostic and follow-up tools as well as treatments are increasingly available in DEing/TrE, cost reimbursement is the main barrier to their widespread utilization in these countries. It is important to keep in mind that even though the cost of genetic analysis and IL-CL is similar in DEing/TrE and DEed, in fact the gross domestic product is completely different, further enlarging the gap in accessibility. Reimbursement of Procysbi® is still lacking even in many high-income countries, although its use has been associated with better compliance [25]; its benefits to quality of life, social interactions, and school function of patients compared with Cystagon® have also been clearly demonstrated [26]. Its cost is largely superior to Cystagon®, about twenty times higher in DEed (383.15 [264–698] vs. 17.95 [1.75–19] USD$/gram), probably explaining why reimbursement strategy is still insufficient. As for its cost in DEing/TrE, data are missing, but the cost reported by one center of USD$1530/gram clearly explains its poor availability in low-income countries.

Another positive result is the emergence of multidisciplinary programs for patients with NC, as well as transition programs from pediatric to adult care, not only in DEed but also in DEing/TrE, even though the proportion of such programs is significantly different. It is indeed remarkable to note that countries which do not have easy access to vital treatments nevertheless manage to organize transition and multidisciplinary programs to improve the global management and the quality of follow-up of their patients. As for DEed, we probably have an incomplete view of the implementation of such adjunctive programs, which are of major importance for patients’ quality of life [27], but may be underdeveloped due to financial and logistical considerations. Nevertheless, the development of these programs represents an important future direction for improving the quality of care of NC patients over the next decade, one that should be kept in mind in DEed.

While innovative research focuses on early diagnosis and better treatment strategies for NC [24, 28, 29], it is essential that these novel tools are not reserved solely for DEed. For example, neonatal screening is now considered one of the most efficient strategies to identify the disease and initiate treatment as early as possible, which could delay the onset of kidney failure and provide optimized care for NC patients. A recent study from Germany showed that starting cysteamine soon after birth yielded almost normal growth and kidney function in these patients with a neonatal diagnosis [30, 31]. Still, the availability of such strategies in low-income countries is questionable, let alone access to potential innovative therapies such as stem cell transplantation [32], or inflammation-targeted therapies [33], which are very likely to be available only in a restricted number of DEed countries.

Despite an increased number of responding centers and countries as compared to 2011, our survey has several limitations. First, the mailing lists we used to distribute the study might be incomplete and might have failed to reach some nephrologists worldwide. Indeed, we are missing data from many countries, in particular coming from Africa, parts of Oceania, Central and South East Asia, and Central and South America, limiting the extrapolation of our results, and probably underestimating the gap with DEed. Moreover, even though a significant number of centers from the USA responded, they are essentially located on the East coast, likely not representing the exact picture of cystinosis in the USA notably for the transition programs of some major centers (as a reminder, we did not have any data from the USA in the 2011 survey). This obviously limits the extrapolation of our results, likely underestimating the number of adult living NC patients, and the implementation of multidisciplinary and transition from pediatric to adult-care programs.

Second, due to ethical reasons and the design of the survey, we did not get any patient data, and therefore could not compare all items between the 2011 and 2022 surveys. Last, it would have been interesting to have a response rate for the survey depending on the type of country. Unfortunately, it is impossible to calculate it in a reliable way since we sent the survey through many different mailing lists, through patient associations (so that they can contact physicians directly), and we presented it during international conferences with a dedicated QR code during the talks.

Despite the fact that the current study clearly unmasks persistent inequalities between DEed and DEing /TrE, this might have still been underestimated at the global level due to limited information received from the African continent. After directly contacting some African centers, we were answered that their lack of resources did not even allow them to diagnose cystinosis, let alone to treat these patients. The genetic background of African patients may account for a lower prevalence of NC as compared to Caucasian populations [34, 35]; still, the demographic expansion that is envisioned in the near future for Africa should encourage the entire international nephrology community to support our African colleagues to gain easier access to diagnosis and treatment for rare kidney diseases. However, the question of reimbursement is crucial and probably the most important limitation to implement these techniques and management in DEing /TrE.

In conclusion, this study documents a significant improvement over the last decade in the availability of genetic and IL-CL testing, and to cysteamine treatment in low-income countries, but also highlights major discrepancies in the management of NC, mainly related to insufficient availability of funds that would allow their access. Indeed, orphan drugs designed to cure rare diseases are often very expensive, due to expensive development costs and the small market size [36]. The recent introduction of orphan drug policies in the USA and in Europe to promote research in the field may help scientific research and clinical progress in DEed, but unfortunately, it did not induce yet a significant reduction in prices. Patients in many countries would benefit from a coherent global rare disease policy ensuring permanent access to diagnostic services and life-saving treatments. We hope that the documentation of international access gaps will help to reduce them by providing objective benchmarking figures that will allow physicians and patient associations to have informed negotiations with their local, regional, and national health authorities.

### Supplementary Information

Below is the link to the electronic supplementary material.

Graphical abstract (PPTX 594 KB)

Supplementary file2 (DOCX 205 KB)

## Data Availability

The data underlying this article will be shared upon reasonable request to the corresponding author.
