# Supplementary material for: Worldwide disparities in access to treatment and investigations for nephropathic cystinosis: a 2023 perspective
Source: Pediatr Nephrol. 2023 Nov 18;39(4):1113–23. doi: 10.1007/s00467-023-06179-3 (PMC10899370; doi:10.1007/s00467-023-06179-3)
Supplement: Supplementary file 1 — Graphical abstract (PPTX 594 KB) [file 467_2023_6179_MOESM1_ESM.pptx]

## Slide 1
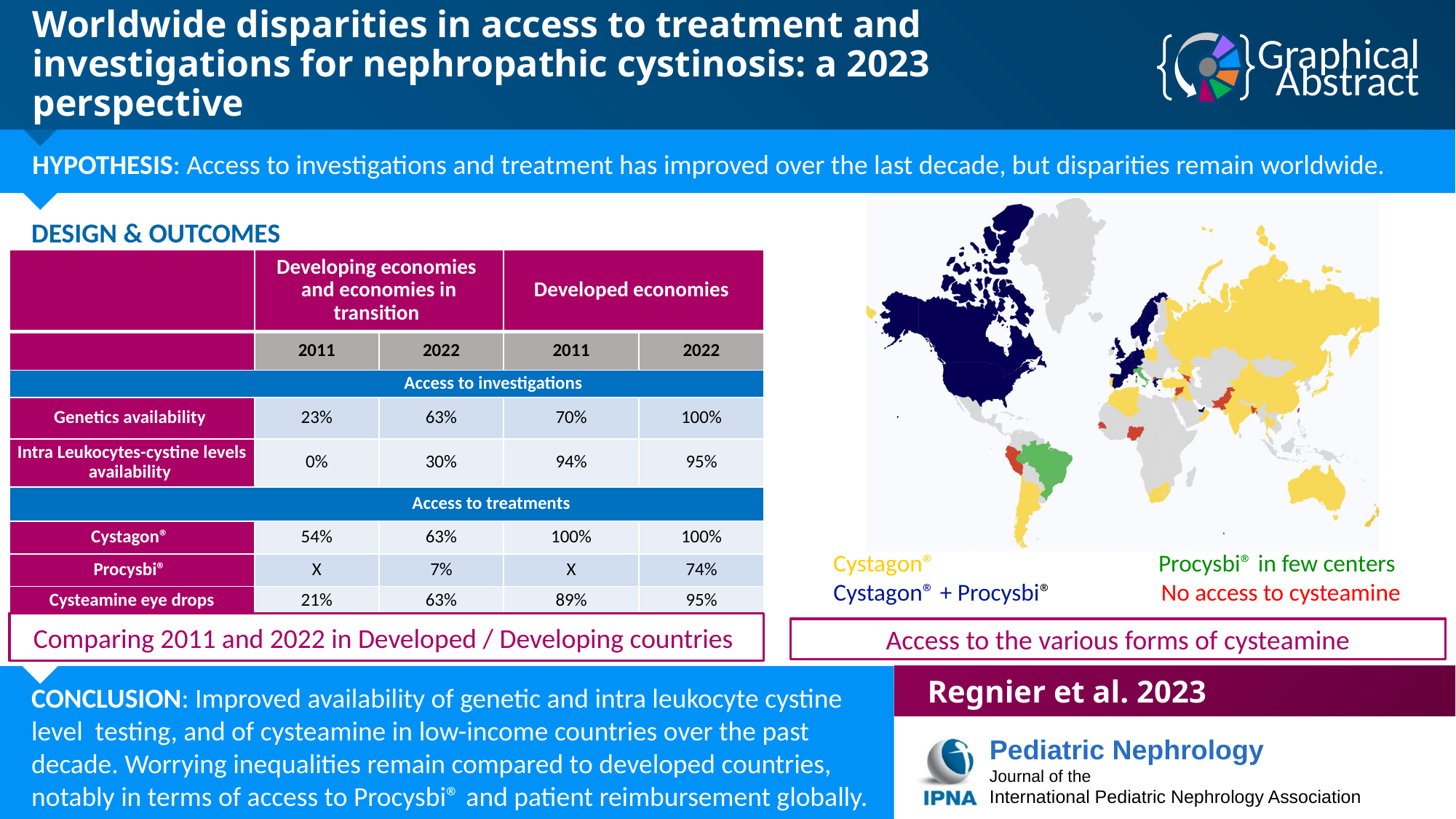

Worldwide disparities in access to treatment and investigations for nephropathic cystinosis: a 2023 perspective
HYPOTHESIS: Access to investigations and treatment has improved over the last decade, but disparities remain worldwide.
Cystagon® Procysbi® in few centers
Cystagon® + Procysbi® No access to cysteamine
DESIGN & OUTCOMES
| | Developing economies and economies in transition | | Developed economies | |
| --- | --- | --- | --- | --- |
| | 2011 | 2022 | 2011 | 2022 |
| Access to investigations | | | | |
| Genetics availability | 23% | 63% | 70% | 100% |
| Intra Leukocytes-cystine levels availability | 0% | 30% | 94% | 95% |
| Access to treatments | | | | |
| Cystagon® | 54% | 63% | 100% | 100% |
| Procysbi® | X | 7% | X | 74% |
| Cysteamine eye drops | 21% | 63% | 89% | 95% |
Comparing 2011 and 2022 in Developed / Developing countries
Access to the various forms of cysteamine
Regnier et al. 2023
CONCLUSION: Improved availability of genetic and intra leukocyte cystine level testing, and of cysteamine in low-income countries over the past decade. Worrying inequalities remain compared to developed countries, notably in terms of access to Procysbi® and patient reimbursement globally.
