# Supplementary material for: Worldwide disparities in access to treatment and investigations for nephropathic cystinosis: a 2023 perspective
Source: Pediatr Nephrol. 2023 Nov 18;39(4):1113–23. doi: 10.1007/s00467-023-06179-3 (PMC10899370; doi:10.1007/s00467-023-06179-3)
Supplement: Supplementary file 2 — Supplementary file2 (DOCX 205 KB) [file 467_2023_6179_MOESM2_ESM.docx]

**Supplementary data**

**Additional file 1: Survey questions**

1- What is your name?

2 - What is your email address?

3 - What is your city of origin?

4 - What is your country of origin?

5 - You are:

- A paediatric nephrologist
- An adult nephrologist
- Other

6- if "other", please specify:

**Paediatric patients (< 20 years old)**

7- How many cystinosis paediatric patients does your team follow?

8- Among them, how many are on conservative treatment?

9- Among them, how many are on dialysis treatment?

10- Among them, how many are after kidney transplant?

**Adult patients**

11- How many cystinosis adult patients does your team follow?

12- Among them, how many are on conservative treatment?

13- Among them, how many are on dialysis treatment?

14- Among them, how many are after kidney transplant?

**Genetics**

15 - Can you perform the genetic analysis to confirm the diagnosis?

- Yes
- No

16 -If you can perform genetic analysis, what is the prize per patient? Please specify currency (euros, dollars, pounds...). If you don't know please write "I don't know"

17 - If you can perform genetic analysis, is it reimbursed to your patient?

- Completely, it does not cost anything for the patient
- Partly, it costs a certain amount for the patient
- Not at all, it is 100% paid by the patient

**Intra-leucocytes cystine levels**

18 - Can you perform measurement of intra-leucocyte cystine levels?

- Yes
- No

19 - If you can perform measurements of intra-leucocyte cystine levels, what is the prize per patient and per sample ? (specify currency). If you don't know, please write "I don't know".

20 - If you can perform measurements of intra-leucocyte cystine level, is it reimbursed to your patient?

- Completely, it does not cost anything for the patient
- Partly, it costs a certain amount for the patient
- Not at all, it is 100% paid by the patient

21 - If you can perform measurements of intra-leucocyte cystine level, why do you perform them?

- To confirm the diagnosis
- To follow patients on cysteamine therapy
- both

**Treatments**

22 - Which oral cysteamine formulation is available in your country?

- Immediate-release (IR) cysteamine (Cystagon®)
- Delayed- release (DR) cysteamine (Procysbi®)
- None
- Both
- Other

23 - If other formulation is available please specify a form.

24 - If IR cysteamine (Cystagon®) is available in your country, what is the price per gram ? (please specify currency)

25 - If IR cysteamine (Cystagon®) is available in your country, is it reimbursed to your patient ?

- Completely, it does not cost anything for the patient
- Partly, it costs a certain amount for the patient
- Not at all, it is 100% paid by the patient

26 - If DR cysteamine (Procysbi®) is available in your country, what is the price per gram ? (please specify currency)

27 - If DR cysteamine (Procysbi®) is available in your country, is it reimbursed to your patient ?

- Completely, it does not cost anything for the patient
- Partly, it costs a certain amount for the patient
- Not at all, it is 100% paid by the patient

28 - If other formulation of cyteamine is available in your country, what is the price per gram ? (please specify currency)

29 - If other formulation of cysteamine is available in your country, is it reimbursed to your patient ?

- Completely, it does not cost anything to the patient
- Partly, it costs a certain amount to the patient
- Not at all, it is 100% paid by the patient

30 - If both are available, which one do you mainly use?

- IR cysteamine (Cystagon®)
- DR cysteamine (Procysbi®)
- Both

31 - If oral cysteamine is available, how do you adapt the daily dose?

- Based on intra-leucocyte cystine level
- Based on patient’s body surface
- Based on prices consideration
- Other

32 - Which cysteamine formulation can you use for eye treatment?

- Cystadrops®
- Cystaran®
- Other
- None

33 - if other, please specify.

34 - If ocular cysteamine is available in your country, what is the prize per vial ? please specify currency

35 - If ocular cysteamine is available in your country, is it reimbursed to your patient ?

- Completely, it does not cost anything for the patient
- Partly, it costs a certain amount for the patient
- Not at all, it is 100% paid by the patient

36 - In your country, do cystinosis patients have an easy access to hemodialysis?

- Yes
- No

37 -In your country, do cystinosis patients have an easy access to peritoneal dialysis?

- Yes
- No

38 -In your country, do cystinosis patients have an easy access to renal transplantation?

- Yes
- No

39 - In your center, do you have a dedicated program for transition from childhood to adulthood care?

- Yes
- No

40 - In your center, do you have a dedicated multi-disciplinary approach or clinic for teenagers and adults with nephropathic cystinosis?

- Yes
- No

41 - If growth hormone is available in your country, what is the price? please specify currency.

42- If growth hormone is available in your country, is it reimbursed to your patient?

- Completely, it does not cost anything for the patient
- Partly, it costs a certain amount for the patient
- Not at all, it is 100% paid by the patient

43 - Which type of the health care insurance is available for Cystinosis patients in your country?

- Public
- Private
- Both

**Supplementary Table 4 : Additional survey answers**

| **Variables** | | **Developing economies  and economies in transition** | | **Developed economies** | | **p value** |
| --- | --- | --- | --- | --- | --- | --- |
|  | | **N** | **N (%)** | **N** | **N (%)** |  |
|  |  | **29** |  | **19** |  |  |
| **Health  care insurance** | Public | 10 | 34.5 | 8 | 42.1 | 0.76 |
|  | Both | 13 | 44.8 | 10 | 52.6 | 0.77 |
|  | Private | 1 | 3.4 | 1 | 5.3 | 0.65 |
|  | No response | 5 | 17.2 | 0 | 0 | NA |
| **Cysteamine oral formulation** | Other form than **Cystagon**® or **Procysbi** ® | 2 | 6.9 | 0 | 0 | 0.51 |
| **Reimbursement** | Total | 1 | 50 | 0 | 0 | NA |
|  | Partial | 0 | 0 | 0 | 0 | NA |
|  | None | 1 | 50 | 0 | 0 | NA |
| **Growth Hormone** |  | | | | | |
| **Reimbursement** | Total | 9 | 34.6 | 17 | 89.5 | **P=0.0002** |
|  | Partial | 7 | 26.9 | 2 | 10.5 | P=0.26 |
|  | None | 10 | 38.4 | 0 | 0 | **P=0.002** |

**Supplementary Figure 4: Cysteamine eye drops formulation available around the world**


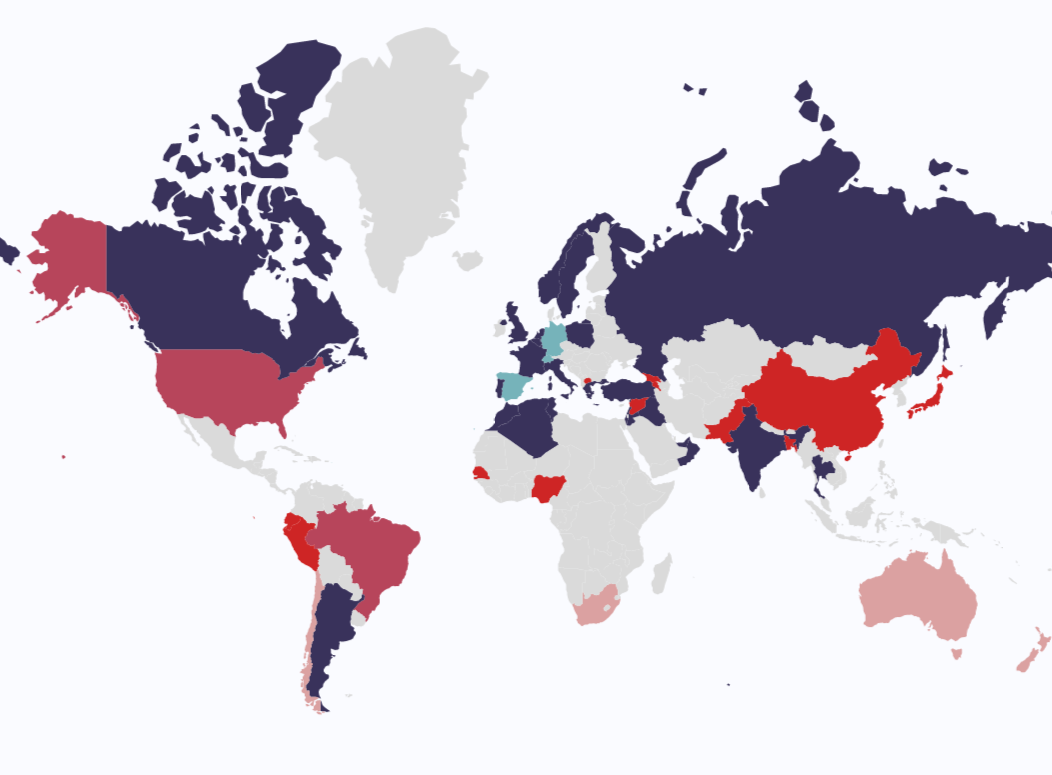


In purple, countries with access to cystadrops **®**. In dark pink, countries with access to both cystadrops **®** and cystaran**®**. In light pink, countries with magistral formulation. In blue, countries with access to both cystadrops **®** and magistral formulation. In red, countries where cysteamine eye drops formulation isn’t available. In grey, non-responding countries.
